# Auroral streamer and its role in driving wave-like pre-onset aurora

**DOI:** 10.1186/s40562-017-0075-6

**Published:** 2017-04-11

**Authors:** Zhonghua Yao, Z. Y. Pu, I. J. Rae, A. Radioti, M. V. Kubyshkina

**Affiliations:** 1 Laboratoire de Physique Atmosphérique et Planétaire, STAR institute, Université de Liège, Liège, Belgium; 2 School of Earth and Space Sciences, Peking University, Beijing, China; 3 UCL Mullard Space Science Laboratory, Dorking, RH5 6NT UK; 4 Physics Faculty, St. Petersburg State University, St. Petersburg, Russia

## Abstract

The time scales of reconnection outflow, substorm expansion, and development of instabilities in the terrestrial magnetosphere are comparable, i.e., from several to tens of minutes, and their existence is related. In this paper, we investigate the physical relations among those phenomena with measurements during a substorm event on January 29, 2008. We present conjugate measurements from ground-based high-temporal resolution all-sky imagers and in situ THEMIS measurements. An auroral streamer (north–south aligned thin auroral layer) was formed and propagated equatorward, which usually implies an earthward propagating plasma flow in the magnetotail. At the most equatorward part of the auroral streamer, a wave-like auroral band was formed aligning in the east–west direction. The wave-like auroral structure is usually explained as a consequence of instability development. Using AM03 model, we trace the auroral structure to magnetotail and estimate a wavelength of ~0.5 *R*_E_. The scale is comparable to the drift mode wavelength determined by the in situ measurements from THEMIS-A, whose footpoint is on the wave-like auroral arc. We also present similar wave-like aurora observations from Cassini ultraviolet imaging spectrograph at Saturn and from Hubble space telescope at Jupiter, suggesting that the wave-like aurora structure is likely a result of fundamental plasma dynamics in the solar system planetary magnetospheres.

## Background

Substorm is a major mode to release the energy in the night-side magnetosphere, which has a consequence of disturbances, including the magnetic field dipolarization in the magnetotail, particle injection in the geosynchronous orbits, high-latitude geomagnetic field perturbations, and explosively auroral intensifications (Frey et al. 2010; Kamide and Brekke 1975; Liou et al. 2001). The mechanism of substorm expansion onset has been a challenging topic for the past half century (Akasofu 1964; Baker et al. 1996; Hones 1979; Lui 1991). As more and more high-quality ground-based and in situ observations became available, particularly the multi-probe missions Cluster (Escoubet et al. 1997), THEMIS (Angelopoulos 2008) and ground-based high-temporal resolution auroral stations (Mende et al. 2007) in the past two decades, it is now widely accepted that bursty bulk flows (BBFs) play an important role in triggering substorm expansions and developing substorm current systems (Angelopoulos et al. 1999, 2008; Birn and Hesse 2014; Yao et al. 2012). On the other side, near-earth instabilities have been confirmed to be a common feature at the beginning of substorm expansion phase (Kalmoni et al. 2015; Liang et al. 2010; Lui et al. 2008a; Nishimura et al. 2016; Rae et al. 2009). It is thus very likely that both the reconnection outflows and near-earth instabilities are essential in triggering substorms. Based on the different triggering mechanisms in substorm expansion onsets, two privileged substorm models have been proposed. The one triggered by magnetotail reconnection is referred to as near-earth neutral line (NENL) model (Baker et al. 1996; Baumjohann 2002; Hones 1979), and the other one driven by near-earth instabilities is referred to as near-earth current disruption (NECD) model (Lui 1991). Moreover, Murphy et al. (2014) suggested that a substorm may be initiated simultaneously by reconnection and near-earth instabilities.

Although near-earth instability and mid-tail magnetic reconnection are usually treated as two individual processes in previous literature, we also notice that there are many common features between the two processes. (1) As shown previously in the literature, near-earth instabilities and reconnection usually take place in thin current sheet conditions (Büchner and Kuska 1999; Drake et al. 1994; Nakamura et al. 2006; Schindler and Birn 1993, 1999). (2) It is also often reported that both reconnection and plasma instability are associated with magnetic dipolarization (Angelopoulos et al. 2008; Lui et al. 2008b; Yao et al. 2013b, 2015); (3) Reconnection and reconnection outflows may also directly trigger a pseudo-substorm (or very small substorm) (Pu et al. 2010; Yao et al. 2014).

The north–south aligned auroral thin arc is often observed and named as auroral streamer, which is usually interpreted as an ionospheric phenomenon of earthward bursty bulk flow (BBF) in the magnetotail (Nishimura et al. 2010, 2011; Sergeev et al. 1996, 2004). In the recent years, as a major benefit from the development of high-temporal resolution ASIs, details of auroral evolution have been dramatically improved. The relation between auroral streamer and substorm onset also becomes a hot research topic for the past few years, and most likely the auroral streamer evolution is consistent with NENL model (Ebihara and Tanaka 2016; Nishimura et al. 2011). However, we need to point out that even though the streamer-like aurora is usually observed ahead of a substorm expansion onset, it does not mean that the streamer triggers the substorm expansion onset. Besides, there are also many substorm onsets not preceded by an auroral streamer. So the relation between BBF and substorm onset is still an open question in our mind.

In this letter, based on the measurements from ground ASIs and in situ THEMIS spacecraft for an auroral event on January 29, 2008, we propose a synthetic model that explains the relation among reconnection outflow (auroral streamer), near-earth instability (wave-like auroral arc), and substorm onset (a small one in this paper). We also analyzed the instability from the scale of the observed beading aurora and the in situ parameters.

## Observations

### Observation of aurora

**Fig. 1:**
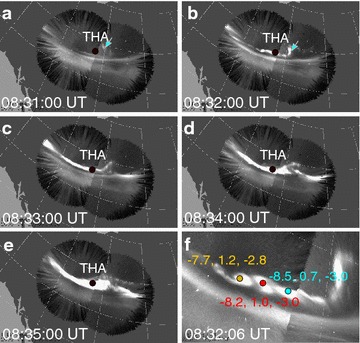
Time sequences of auroral activities from ASIs [the left station is Fort Simpson (FSIM), and the right station is Fort Smith (FSMI)]. The *black dot* in **a**–**e** is the footpoint of THEMIS-A from AM03 model. **f** The zoom-in auroral snapshot at 08:32:06 UT; the *yellow*, *red*, and *blue* dots are identified by eye to represent the locations of three identical auroral patches

Figure 1 shows the auroral imagers for every minute from 08:31 UT to 08:35 UT. The footpoints of THEMIS-A are given by AM03 model (Kubyshkina et al. 2002, 2009, 2011), which aims to provide accurate mapping results during substorm expansions. At 08:31:00 UT (shown in Fig. 1a), a north–south auroral streamer appears, which is located at the poleward of THEMIS-A. Electrons are accumulated on the dusk flank of a BBF, which precipitate into ionosphere and form aurora (Sergeev et al. 1996). Usually, the north–south auroral structure is considered as an ionosphere phenomenon of earthward BBF (Nishimura et al. 2011; Ohtani 2004; Sergeev et al. 2000). The north–south auroral streamer might be related to a mid-tail earthward BBF detected by THEMIS-C at [−18.4, −2.1, −5.9] *R*_E_ in Geocentric solar magnetospheric (GSM) coordinates (not shown in this paper). Two faint parallel auroral arcs are identified, which we name as ARC-P (poleward) and ARC-E (equatorward). In Fig. 1b (08:32:00 UT), the streamer structure clearly extended to ARC-P; meanwhile, ARC-P arc became illuminant, presenting small-scale wave-like structures. In Fig. 1c–e, ARC-P was step-by-step further intensified. A major auroral intensification occurs a few minutes later, which will be discussed in the next section.

Figure 1f shows the details of wave-like structure of ARC-P at 08:32:06 UT. We can easily identify by eye the periodic illuminations from the auroral image, and we use the colored dots to represent the periodic brightness. The geographic longitudes and latitudes of these colored dots are [239.8, 61.7], [241.5, 61.6], and [243.2, 61.4]. With AM03 model, we are able to trace these spots to magnetotail neutral sheet. The locations are [−7.71, 1.23, −2.85] *R*_E_, [−8.16, 0.98, −2.96] *R*_E_, and [−8.48, 0.66, −3.04] *R*_E_ in Geocentric solar magnetospheric (GSM) coordinates. The separations between the nearest two spots over the three red spots are 0.45 *R*_E_ and 0.52 *R*_E_. We need to point out that the identification by eye is not very accurate, and the AM03 mapping to magnetotail is not accurate. As we just need to roughly estimate a scale of the wavelength, we thus use 0.5 *R*_E_ to represent the wave length in this paper. The inaccuracy does not seriously affect our main conclusion.

### In situ measurements

**Fig. 2:**
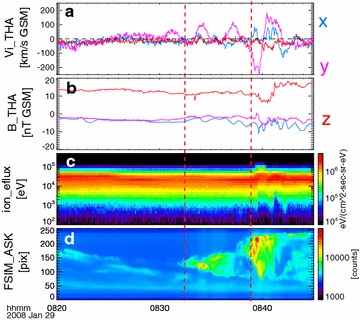
Overview of THEMIS-A measurements between 08:20 UT and 08:50 UT. **a** Ion bulk flow components in GSM coordinates, **b** vector magnetic field components in GSM coordinates, **c** ion differential energy flux, and **d** auroral keogram of FSIM station. The two *dashed vertical red lines* indicate two auroral intensifications

Figure 2 shows in situ measurements of ion bulk velocity (Fig. 2a), magnetic field (Fig. 2b), and ion energy spectrum (Fig. 2c) from THEMIS-A spacecraft, located at [−8.7, 0.7, −3.0] *R*_E_ in GSM coordinates. We have combined the ion measurements from the electrostatic analyzer (ESA) (McFadden et al. 2008) and the solid-state telescope (SST) (Angelopoulos 2008). The magnetic field measurements are from the fluxgate magnetometer (FGM) (Auster et al. 2008). A significant decrease of the magnetic component *B*_z_ was observed at ~08:39 UT, followed by a significant increase within 2 min. We do not speculate a mechanism for the major intensification at ~08:39 UT. From the auroral keogram, we notice that the major intensification was at a higher latitude, so it is very likely that the source of the aurora intensification at ~08:39 was in a higher latitude, and thus the near-earth spacecraft observed a dipolarization after the auroral intensification. A clear ion energization process, as shown in Fig. 2c, accompanies the quick change in the magnetic component *B*_z_. The auroral keogram (Fig. 2d) shows two intensifications (the two vertical dashed red lines), one started at ~08:32 UT and the other one at ~08:39 UT. The latter one is the major intensification, which expanded to lower and higher latitudes. The two-step auroral intensification is also previously reported (Pu et al. 2010). The auroral time sequences in Fig. 1 describe the earlier intensification in Fig. 2d. During the earlier auroral intensification (08:32 UT–08:37 UT), magnetic field shows periodic variation and the ion bulk velocity shows pulsating enhancements toward duskward. It is noteworthy that a dawnward flow was observed by THEMIS-A between 08:39 UT and 08:40 UT, which is likely a consequence of flow braking/diversion in the near-earth magnetotail (Shiokawa et al. 1997). Flows are diverted to both dawn and dusk sides, which are considered to be associated with a substorm current wedge. For example, Birn et al. (2004) and Keiling et al. (2009) show that the flow diversion at both dawn and dusk sides could form two flow vortices, which drive a pair of field-aligned current and form a substorm current wedge.

**Fig. 3:**
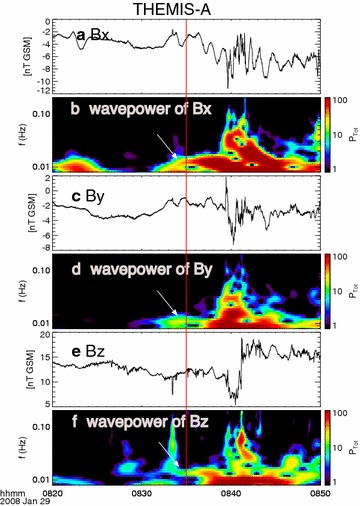
Wavelet analysis of the vector magnetic field components. The *red line* marks the time 08:35 UT

Figure 3 shows the wavelet analysis results of the 0.25-s time resolution magnetic field components. Identical wave power in the frequency range of 0.01–0.02 Hz occurs in all three components at ~08:32 UT (the white arrows), consistent with the wave-like auroral structure. This discrete wave power lasts until ~08:38 UT, followed by a broaden frequency intensification. Most likely, the wave intensification between 08:32 UT and 08:38 UT is related to the first auroral intensification (08:32 UT–08:37 UT) as shown in Fig. 2d.

## Discussion

In the aurora breakup event on January 29, 2008, an auroral streamer was formed at ~08:31 UT. THEMIS-C has also observed an earthward BBF event at ~08:31:30 UT. It is very likely that the two signatures are physically related. About 1 min later, the streamer reached the equatorward auroral arc ARC-P and led to an intensification on ARC-P, accompanied by an azimuthally aligned wave-like perturbation. THEMIS-A, whose footpoint was on ARC-P, has detected a duskward low-speed bulk velocity. Wavelet analysis of the magnetic field from THEMIS-A shows a frequency of 0.01–0.02 Hz. We thus estimate a wavelength with *λ*_y_ = *V*_y_ · *T*_period_, as performed in previous literature (Saito et al. 2008). In our estimation, *T*_period_ ~ 50–100 s and *V*_y_ ~ 42 km/s (average between 08:30:30 UT and 08:31:30 UT), so the wavelength estimated from THEMIS-A in situ measurements should be 2100–4200 km, which is very consistent with that determined from the ground aurora, i.e., ~0.5 *R*_E_. We adopt the average drifting velocity between 0.5 min and 1.5 min prior to the ground wave-like aurora (08:32:06 UT), as we have taken into consideration the Alfvén transit time between magnetosphere and ionosphere, which is usually tens of seconds to 2 min (Keiling et al. 2009; Lui et al. 2010; Yao et al. 2013a).

Generally, our event is consistent with the picture in Nishimura et al. (2011) that is based on a statistical study of auroral breakup with ASIs. They proposed a model that auroral streamer initiated from poleward boundary propagates equatorward and eventually triggers a substorm expansion at the pre-onset auroral arc. Moreover, we present a quantitative analysis of the wave-like structure that appears immediately after the arrival of auroral streamer structure. We estimated the wavelength of the wave-like structure in the magnetotail with AM03 model. The result is highly consistent with the azimuthal wavelength of the drift instability determined from THEMIS-A in situ measurements.

Both the in situ analysis and ground auroral imagers have shown that the wavelength of the instability mode is ~0.5 *R*_E_. The consistence between the drifting wave length determined from THEMIS-A and ground auroral wavelength (mapped to magnetosphere) strongly implies that this wave-like structure is a drift wave mode. In the inner edge plasma sheet, ballooning mode instability (Ohtani and Tamao 1993; Pritchett and Coroniti 1999; Pu et al. 1999) and mirror mode instability (Rae et al. 2007) are likely to be excited. We have also checked the mirror mode instability threshold and found that the plasma environment in our event is not favorable to mirror mode instability, as the ion anisotropy was not significant in our event. Most likely, this wave-like auroral structure is associated with a ballooning-like instability in the near-earth magnetotail.

Here we estimate the ion gyroradius of ~1500 km with in situ measurements (*B* ~ 15 nT and *E* ~ 25 keV), which is a half of the wavelength of the ballooning mode instability. Therefore, the kinetic effect needs to be considered in our analysis. Pu et al. (1997) carried out a general analysis of the magnetohydrodynamics (MHD) ballooning mode, and they found that a bulk flow could enhance the growth of ballooning mode. In our event, from the aurora imagers, the wave-like structure was formed at the arrival of auroral streamer, which is consistent with Pu et al. (1997)’s flow enhancing ballooning theory, and moreover the wave-like structure did not evolve to a major substorm event; we suggest that this is because ion kinetic effect limited the development of ballooning instability, as suggested by Pritchett and Coroniti (1999). They found that the ballooning instability evolves toward shorter wavelengths and stops at the ion gyroradius scale.

**Fig. 4:**
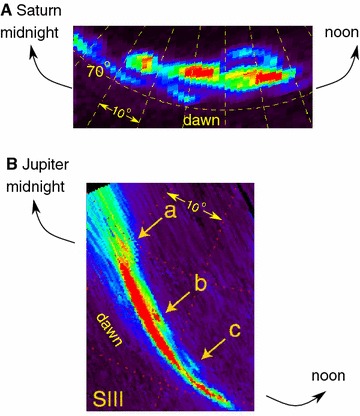
**A** The auroral image of Saturn’s polar region from Cassini UVIS at 21:49 UT on May 20, 2013, adopted from Radioti et al. (2016). **B** The auroral image of Jupiter’s polar region from HST at 05:37 on March 5, 2007 (Adopted from Radioti et al. (2008))

Similar wave-like structures also exist in other planetary magnetospheres. For example, Radioti et al. (2016) present an auroral breakup event at Saturn with Cassini UVIS (Fig. 4A). The authors reported on wave-like structures in the dawn sector, which co-rotate with Saturn, and related them to plasma flows enhanced from magnetic reconnection. The aurora at Jupiter has shown similar morphological features. Radioti et al. (2008) reported on polar spots (a, b, and c on Fig. 4B) located in the polar dawn sector based on Hubble space telescope measurements. The analogy between Earth and Saturn/Jupiter implies that fundamental plasma dynamics are shared among the solar system planets.

## Conclusion

Using data from THEMIS and ground-based aurora stations, we analyze the physics of the pre-onset beading aurora structure and its potential role in driving substorm expansion. We also reveal that similar process may exist at other planetary magnetospheres (i.e., Saturn and Jupiter) from the auroral imagers taken by Cassini and HST. The main results are summarized as follows:

1. Wave-like auroral structure develops on the pre-existing auroral arc ARC-P at the arrival of a streamer to this arc.
2. We estimate a wavelength of ~0.5 *R*_E_ from both the magnetosphere mapping results of auroral imagers and the in situ THEMIS-A measurements.
3. As suggested in previous literature (Pritchett and Coroniti 1999), the ballooning instability can develop in a convectively driven plasma sheet, but does not continue growing when the wavelength is as small as ion gyroradius scale. In our event, the wavelength is at the scale of the ion gyroradius, and the aurora was not developed to a major breakup until ~6 min later. We thus suggest that the major intensification is not a direct consequence of the ballooning instability, which is consistent with the theory of Pritchett and Coroniti (1999).
4. We also show similar wave-like structure at Saturn’s polar region with the aurora measurements from Cassini UVIS, and at Jupiter’s polar region with HST measurements. The similarity between terrestrial and Saturnian/Jovian auroral arcs suggests that the ballooning-like instability might be a fundamental process in magnetosphere–ionosphere coupling in solar system planets.

## Abbreviations

ASIs: all-sky imagers
BBF: bursty bulk flow
NENL: near-earth neutral line model
NECD: near-earth current disruption model
MHD: magnetohydrodynamics
GSM: Geocentric solar magnetospheric
UVIS: ultraviolet imaging spectrograph
HST: Hubble space telescope
